# Regulation of muscle stem cell fate

**DOI:** 10.1186/s13619-022-00142-7

**Published:** 2022-12-02

**Authors:** Xin Fu, Cheng-le Zhuang, Ping Hu

**Affiliations:** 1 Spine Center, Xinhua Hospital Affiliated to Shanghai Jiao Tong University School of Medicine, Shanghai, 200092 China; 2 Colorectal Cancer Center/Department of Gastrointestinal Surgery, Shanghai Tenth People’s Hospital Affiliated to Tongji University, Shanghai, 200072 China; 3 Guangzhou Laboratory, Guanghzou International Bio Lsland, No. 9 XingDaoHuan Road, Guangzhou, 510005 China; 4 Institute for Stem Cell and Regeneration, Chinese Academy of Sciences, Beijing, 100101 China

**Keywords:** Muscle stem cells, Skeletal muscle regeneration, Asymmetric division, MuSC heterogeneity, Microenvironments, Transcription regulation

## Abstract

Skeletal muscle plays a critical role in human health. Muscle stem cells (MuSCs) serve as the major cell type contributing to muscle regeneration by directly differentiating to mature muscle cells. MuSCs usually remain quiescent with occasionally self-renewal and are activated to enter cell cycle for proliferation followed by differentiation upon muscle injury or under pathological conditions. The quiescence maintenance, activation, proliferation, and differentiation of MuSCs are tightly regulated. The MuSC cell-intrinsic regulatory network and the microenvironments work coordinately to orchestrate the fate transition of MuSCs. The heterogeneity of MuSCs further complicates the regulation of MuSCs. This review briefly summarizes the current progress on the heterogeneity of MuSCs and the microenvironments, epigenetic, and transcription regulations of MuSCs.

## Background

Skeletal muscle accounts for about 40% of body mass and 50–75% of body proteins in healthy humans (Frontera and Ochala 2015). Healthy skeletal muscle is critical for physiological functions such as locomotion, breathing, metabolism, energy and protein storage, and immune regulation. Better muscle function will significantly improve life quality in humans. Skeletal muscle is a tissue with relatively high regeneration ability to repair everyday wear and tear and other mild injuries. Skeletal muscle regeneration is the key to maintaining working skeletal musculature both under normal conditions and upon injury. The failure of skeletal muscle regeneration renders locomotion deficiency, metabolism defects, and lethality.

Muscle stem cells (MuSCs) are adult stem cells residing in skeletal muscle, and they are the primary workforce to regenerate and maintain the muscle tissue integrity. The activity of MuSCs is subjected to highly choreographed regulation during the muscle regeneration process. The microenvironments of MuSCs in both intact and injured muscles have been shown to have important roles in sending the information to guide the activity of MuSCs. A well-knitted cell-intrinsic regulatory network responds to the microenvironment cues and determines the cell fate conversion of MuSCs. The epigenetic and transcription regulatory armamentarium is critical to the cell-intrinsic network controlling MuSC fate transition (Fu et al. 2021). Here, the recent advances in the microenvironments, epigenetic, and transcription regulation of MuSCs are briefly reviewed.

## MuSCs and skeletal muscle regeneration

**Fig. 1:**
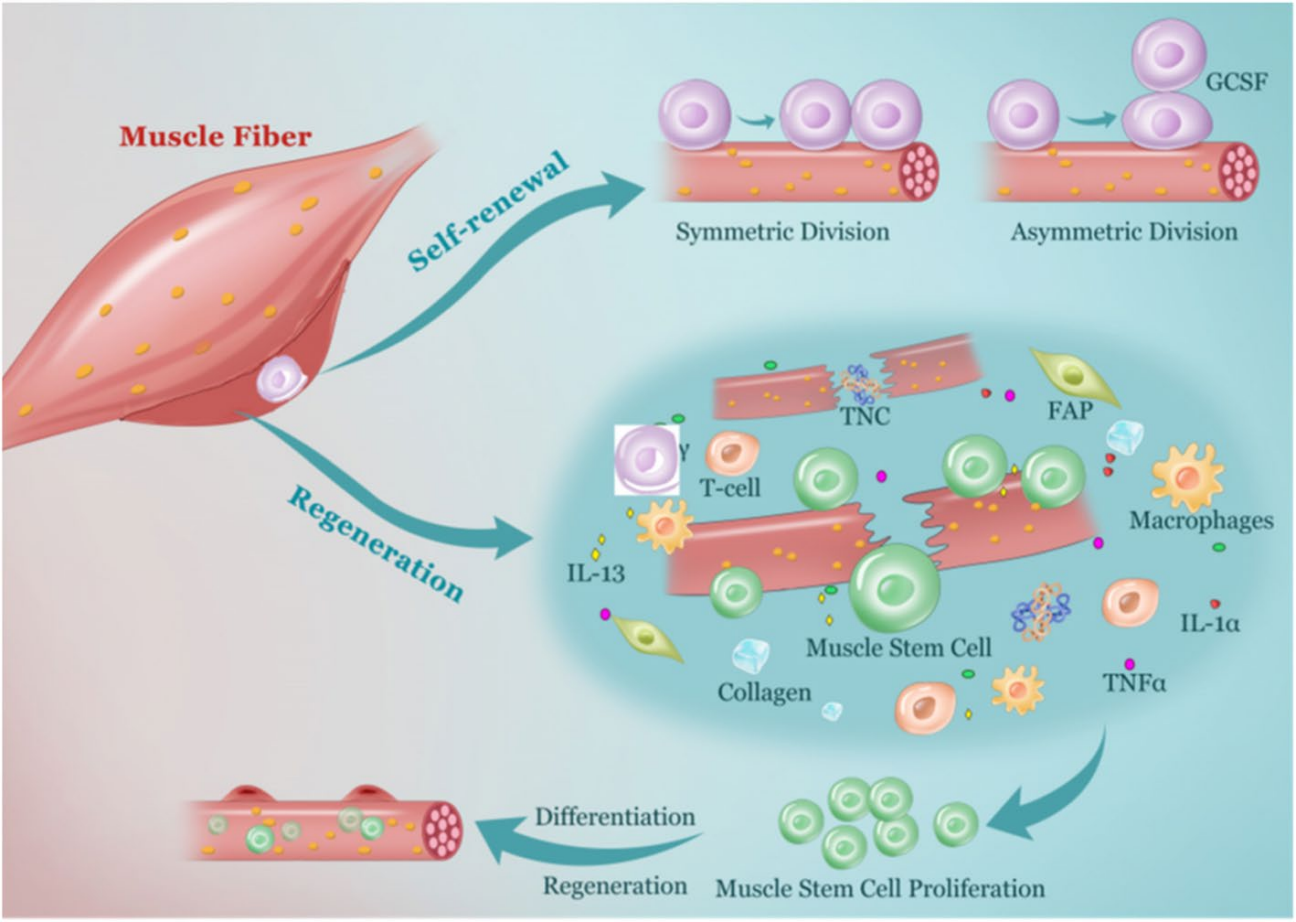
The life cycle of muscle stem cells. MuSCs are located between the basal lamina of the muscle fibers and the sarcolemma. They undergo symmetric division or asymmetric division to maintain muscle homeostasis. G-CSF and EGFR have important roles in regulating the asymmetric division of MuSCs. Upon injury, MuSCs are activated and released from the niche. The necroptotic myofibers, FAPs, and infiltrated immune cells provide the microenvironment for MuSC proliferation. TNFα, IFNγ, IL-1ꞵ, IL-13, TNC, Collagen V, and other factors facilitate MuSC proliferation. The proliferated MuSCs fuse with the existing myofibers or differentiate to new myofibers to regenerate injured skeletal muscle. A small portion of MuSCs return to quiescence and replenish the stem cell pool

MuSCs were initially identified in 1961 by Alexander Mauro and referred to as satellite cells due to their locations around the myofibers (Mauro 1961). Different from the multinucleated myofibers, MuSCs are mononucleated unipotent adult stem cells. These cells locate between the sarcolemma and the basal lamina of muscle fibers (Fig. 1). MuSCs have been identified in amphibian, reptilian, aves, and mammals (Ishikawa 1966, Lipton and Schultz 1979, Rupik et al. 2012, Tanaka et al. 2016, Yorita et al. 1980). Their ability to support muscle regeneration is conserved in all vertebrates (Hartley et al. 1992, Kahn and Simpson 1974, Popiela 1976).

MuSCs display great abilities to support muscle regeneration. The engrafted MuSCs go through active expansion for approximately tenfold after transplantation (Collins et al. 2005). And 7 engrafted MuSCs can regenerate over 100 myofibers containing 25, 000—30, 000 nuclei (Collins et al. 2005). The disruption of MuSC function under aging, muscle dystrophy, and other pathological conditions, leads to muscle regeneration defects, as reviewed in many publications (Yamakawa et al. 2020).

## Quiescent MuSCs

At the neonatal stage, skeletal muscle undergoes a wave of significant mass gain. It is mainly supported by MuSCs. At this stage, MuSCs undertake massive expansion to support the rapid growth of skeletal muscle. Most of the expanded MuSCs differentiate by fusing with the existing myofibers. The small portion of the expanded MuSCs remain to be undifferentiated and enter quiescence by staying at G0 stage (Bachman et al. 2018, Cheung et al. 2012). Compared to the skin, intestine, blood system, and other fast turnover tissues, skeletal muscle shows a relatively slower turnover rate, and the average life of human muscle cells is measured by years. To adapt to the slow turnover of skeletal muscle, MuSCs remain quiescence for most of the time in adulthood.

Failure to stay in quiescence leads to the loss of stemness, precocious differentiation, senescence, and apoptosis of MuSCs, which results in the decline of MuSC number and activity (Bjornson et al. 2012, Cheung et al. 2012, Evano and Tajbakhsh 2018, Garcia-Prat et al. 2016, Mourikis et al. 2012a, Shea et al. 2010). The disruption of MuSC quiescence is usually associated with aging and muscle diseases. It causes impaired long-term regeneration ability (Jiang et al. 2014).

Quiescent MuSCs have higher engraftment efficiency and are considered to possess the highest stemness (Arjona et al. 2022). The quiescent MuSCs display several characteristic features, such as smaller cell size, higher nucleo-cytoplasmic ratio, lower RNA and protein synthesis level, and mainly perform fatty acid oxidation (Eliazer and Brack 2016, Rodgers et al. 2014, Ryall et al. 2015). They express Pax7, the characteristic transcription factor of MuSCs. The quiescent MuSCs do not express MyoD (Olguin and Olwin 2004). Besides that, the quiescent MuSCs also have unique gene expression profiles compared to the activated MuSCs. Genes such as Calcitonin Receptor (CalcR), CD34, α_7_-Integrin, Sprouty 1, Syndecan-4, CXCR4, Integrinβ1 (ITGB1), M-Cadherin, N-Cadherin, Notch Receptor, Osmotically inducible lipoprotein β (OSMβ), and Teneurin transmembrane protein 4 (Tenm4/Odz4) are highly expressed in quiescent MuSCs (Fu et al. 2015b, Fukada et al. 2013, Goel et al. 2017, Machado et al. 2017a, Machado et al. 2017b, van Velthoven et al. 2017, Yamaguchi et al. 2012).

MuSCs tend to be activated during the isolation procedure in which disruption of the original muscle structure is almost unavoidable. Identifying more quiescent MuSC markers and isolating quiescent MuSCs from skeletal muscle are under intensive investigation. Recently, Tubastatin A, and Histone deacetylase 6 (HDAC6) inhibitor, has been shown to be able to retain MuSCs in quiescent state by preserving primary cilium (Arjona et al. 2022). The quiescent state of MuSCs is actively maintained by the combination of several signaling cascades. Notch signaling takes a central role in MuSC quiescence maintenance. MuSC-specific knockout of Notch2 slightly reduces stem cell number. MuSC-specific double knockout of Notch1 and Notch2 almost depletes quiescent MuSCs completely, suggesting that Notch1 and Notch2 work coordinately to preserve MuSCs at the quiescent stage by preventing spontaneous activation (Fujimaki et al. 2018). Rbpj is the major transcriptional regulator of Notch signaling pathway. Consistent with the critical role of Notch signaling pathway in quiescence maintenance, the quiescent MuSCs display a high expression level of Rbpj expression (Bjornson et al. 2012). MuSC-specific knockout of Rbpj results in the loss of MuSCs (Mourikis et al. 2012b). Notch signaling helps maintain MuSC quiescence by multiple means. Active Notch signaling maintains the expression of Pax7, inhibits MyoD expression, and improves the homing of MuSCs (Evano and Tajbakhsh 2018). Moreover, activation of Notch signaling can also stimulate the expression of Collagen V, which in turn serves as a surrogate ligand to activate CalcR signaling and facilitate the quiescence maintenance (Baghdadi et al. 2018a, Baghdadi et al. 2018b).

Other factors are also involved in quiescence regulation. Extracellular matrix is required for MuSCs to remain in quiescence. In conditional N-cadherin and M-Cadherin knockout mice driven by MyoD-iCre, MuSCs stay at the early transition stage from quiescence to activation (Goel et al. 2017). Wnt4 activates Rho signaling and inhibits Yes-associated protein (YAP) signaling to retain MuSCs at quiescence stage (Eliazer et al. 2019). Cytoskeleton remodeling mediated by the activation of Rac-Rho GTPase switch is required for the activation of quiescent MuSCs. Rho GTPase helps retain MuSCs in quiescent stage, while the switch from Rho to Rac GTPase upon injury marks the early event of quiescence exit (Kann et al. 2022). β-hydroxybutyrate induced by fasting promotes MuSCs going to deep quiescence by preventing HDAC1 mediated de-acetylation of p53 (Benjamin et al. 2022). Forkhead box O (FoxO) transcription factors are critical to maintaining muscle stem cell quiescence. At geriatric age, the level of niche-derived IGF1 increases which activates Akt, and in turn inhibits FoxO activity deteriorating the genuine quiescent stage of MuSCs (Garcia-Prat et al. 2020). Angiopoietin 1 (Ang1) binds its receptor Tie2. The expression level of Tie2 is high in quiescent MuSCs. It inhibits ERK signaling and prevents MuSCs from entering the cell cycle to maintain quiescence (Abou-Khalil et al. 2009). Di-methyltransferase Suv4-20h1 retains the MyoD locus at the nuclear peripheral region and preserves the H3K27me3 associated heterochromatin to maintain the quiescence of MuSCs (Boonsanay et al. 2016). The Ser 51 phosphorylation of translation initiation factor eIF2α represses translation in general in MuSCs to help retain MuSCs at the quiescent stage (Zismanov et al. 2016). Recently, the mechanosensitive Ca^2+^ channel Piezo1 has been reported to involve in MuSC quiescence maintenance. Piezo1 knockout leads to increased reactive oxygen species (ROS) and MuSC senescence and cell death (Peng et al. 2022). Currently, results indicate that the maintenance of MuSC quiescence is tightly regulated by a complex cellular intrinsic network containing transcription factors, translation factors, extracellular matrix, metabolites, and mechano sensors. How these factors are knitted together remains to be explored.

The cell–cell communications between MuSCs and the surrounding cell types also help maintain the quiescent stage of MuSCs. For example, MuSCs secrete Vascular Endothelial Growth Factor A (VEGFA). VEGFA acts on the capillary vascular endothelial cells, which are close approximate to MuSCs, to activate the expression of Notch ligand Dll4. The increased Dll4 level further activates Notch signaling, which is critical to retaining MuSCs in quiescence (Verma et al. 2018). Aging disrupts the quiescence of MuSCs. MuSCs are activated and differentiated precociously. This is one of the reasons for the decline of muscle regeneration ability in aged muscle (Chen et al. 2020). The mechanism of quiescence acquiring and maintenance is under intensive investigation currently. Exploring this mechanism will expand our horizon of understanding stemness.

## Symmetric and asymmetric division of MuSCs

MuSCs are capable of two manners of divisions, namely symmetric and asymmetric division. Using Myf5-Cre: Rosa26R-YFP mice, two types of divisions were observed. In symmetric division, Myf5- MuSCs undergo planar divisions, in which the division orientation is parallel to the basal lamina. Symmetric division generates two identical stem cells. MuSCs can be expanded by symmetric division. In asymmetric division, one Myf5-MuSC undergoes apical-basal division, in which the division orientation is perpendicular to the basal lamina. Asymmetric division produces one Myf5- stem cell at the apical position and one Myf5 + progenitor at the basal position (Kuang et al. 2007). Symmetric division increases MuSC number, while asymmetric division maintains the constant number of MuSCs.

Asymmetric division is not only marked by the perpendicular division orientation, but also characterized by the asymmetric distribution of template DNA and several proteins. The original DNA from the mother cell is inherited by the daughter stem cell; while the newly synthesized DNA is distributed to the more differentiated daughter cells (Conboy et al. 2007). In this division manner, the daughter stem cells always keep the original copy of DNA to maintain the high fidelity of DNA information. Numb endocytic adaptor protein is co-segregated with the original DNA copy to the daughter cells expressing stem cell marker, while the more differentiated daughter cells do not get Numb protein (Rocheteau et al. 2012, Shinin et al. 2006). Similarly, Dystrophin and Mark2 are distributed to the daughter stem cells, but not to the more differentiated daughter progenitor cells (Dumont et al. 2015). MyoD, SCA1, pp38α/β, pERK, and Par complex components such as PKCλ and Par3 specifically segregate to the more differentiated daughter cells (Bernet et al. 2014, Troy et al. 2012). In contrast, these proteins are equally distributed to both daughter cells under symmetric division (Dumont et al. 2015, Troy et al. 2012).

Several signaling pathways have been suggested to regulate asymmetric division versus symmetric division. Epidermal Growth Factor/Epidermal Growth Factor Receptor (EGF/EGFR), Mitogen-Activated Protein Kinase (MAPK), and Protease-activated receptors (Par) signaling are required for asymmetric division (Dumont et al. 2015, Troy et al. 2012, Wang et al. 2019). The activation of EGF/EGFR signaling promotes the asymmetric localization of EGFR in the daughter stem cells (Wang et al. 2019). Similarly, the activation of MAPK signaling is correlated with the asymmetric localization of SDC4, PKCλ, and PAR3 (Troy et al. 2012). The expression of Dystrophin also promotes the asymmetric localization of PAR3 (Dumont et al. 2015). In contrast, Wnt7a promotes symmetric division through non-canonical Wnt signaling pathway. Vangl2, the important component of Planar Cell Polarity (PCP)signaling pathway, which is highly expressed in the more activated MuSCs, is required for Wnt7a induced symmetric division. Wnt7a induces the asymmetric localization of Vangl2 (Le Grand et al. 2009). The polarized localization of these molecules may help MuSCs sense the unequally distributed signaling molecules in the microenvironment and maintain the stem cell property. The different environmental cues that originate from the basal lamina and myofibers may participate in shaping the fate of the two daughter cells. The mechanism for the determination of asymmetric or symmetric division commitment needs more intensive investigations.

In isolated myofibers, the orientation of the most of the divisions is parallel to the long axis of myofibers, suggesting that the majority of MuSCs undergo symmetric division. Depending on the system used, perpendicular divisions account for about 10–35% of dividing cells (Kuang et al. 2007, Siegel et al. 2011). On the damaged “ghost” myofibers in injured muscle, about 80% of the divisions are parallel to the long axis of the myofibers, while less than 10% of the divisions are perpendicular to the long axis of myofibers (Webster et al. 2016), suggesting most of the MuSCs commit symmetric division.

Though the asymmetric division accounts for a relatively rare division manner of MuSCs, it plays an uncomplemented role in muscle homeostatic maintenance and muscle regeneration. In Duchenne muscular dystrophy (DMD), there is a 75% reduction of asymmetric division, which results in decreased rate to generate myogenic progenitors to support muscle regeneration (Dumont et al. 2015, Feige et al. 2018). The loss of balance between the symmetric division and asymmetric division has been considered to be one of the reasons for muscle regeneration defects in old skeletal muscles. In aged muscles, increasing activity of Janus kinase/signal transducer and activator of transcription (Jak-STAT) signaling leads to reduced symmetric division, increased progenitor cell number, and reduced number of stem cells, which in turn has the consequence of declined muscle regeneration ability (Feige et al. 2018, Price et al. 2014, Tierney et al. 2014).

The quiescent MuSCs display heterogeneity. Some MuSCs have high Pax7 expression levels; the others have medium levels of Pax7 expression. The MuSCs with more tendency to differentiate have low Pax7 expression levels. Pax7^High^ MuSCs display a higher capacity for asymmetric division (Rocheteau et al. 2012). Upon quiescence exit, it takes longer for Pax7^High^ MuSCs to enter the first cell cycle (Sutcu and Ricchetti 2018). Whether Pax7^High^ MuSCs represent stem cells with higher stemness remains to be explored. How asymmetric division and symmetric division contribute to the heterogeneity of MuSCs remains to be further explored. Especially the current studies on asymmetric and symmetric division are all based on myofiber culturing. Under the condition of massive myofiber disruption, whether MuSCs still undergo asymmetric division both in vitro and in vivo is still a pending question.

## MuSCs fate conversion after injury

The niche of quiescent MuSCs is disrupted after the occurrence of muscle injury. When the niche is disrupted, quiescent MuSCs lose the protection of the niche and are activated. The activated MuSCs re-enter the cell cycle to proliferate. The morphology of the activated MuSCs changes. The size of the activated MuSCs is larger, and the cytoplasm also expands. The number of mitochondria and other organelles increases (Anderson 2000, Wozniak et al. 2005). The earliest marker for MuSC activation is phosphorylated p38. MyoD expression and the Myf5 protein level increase in activated MuSCs (Jones et al. 2005, Kondoh et al. 2007, Zhang et al. 2010). It takes over 48 h for the activated MuSCs to complete the first cell cycle upon exit of quiescence, which is much longer compared with the normal cycling cells (Marescal and Cheeseman 2020).

After activation, MuSCs undergo fast expansion within a short period. In mouse model, MuSCs mainly proliferate 3–4 days after injury induced by cardiotoxin (CTX) injection (Fu et al. 2015b, Webster et al. 2016). In human, an increasing number of MuSCs at G2/M phase have been observed 48 h after exercise (McKay et al. 2010). The expanded MuSCs differentiate to myoblasts, which do not express Pax7, but the expression of MyoD and Myf5 persists. The myoblasts further differentiate to myotubes. At the end of the regeneration process, the remaining MuSCs return to quiescence and home to the niche to maintain the stem cell reservoir (Collins et al. 2005).

The conversion from MuSC to myotubes in vivo is more complex than the straight path described above. Recent single-cell sequencing results indicate that some of the MuSCs retain stemness during the regeneration process. Both MuSCs and inflammatory cell markers are co-expressed in this subpopulation of MuSC (Oprescu et al. 2020). With more single-cell sequencing data available, more insights into the MuSC fate transition in vivo will be obtained.

## Transcription and epigenetic regulation of MuSC fate transition

Transcription factors are key to regulating the lineage determination and fate conversion of MuSCs (Fu et al. 2021). MuSCs are marked by two paired transcription family members, namely Pax3 and Pax7. They are among the earliest expressed transcription factors at the early MuSC development stage. (Gros et al. 2005, Kassar-Duchossoy et al. 2005, Relaix et al. 2005). Depleting both Pax3 and Pax7 leads to myogenesis arrest at embryonic development and fetal development stages (Relaix et al. 2005).

Pax7 is the key transcription factor for MuSC functions and considered to be the major MuSC marker. Pax7 is able to bind and remodel chromatin structure by reducing the level of H3K4me1 and H3K27Ac to increase the nucleosome-free open chromatin region (Budry et al. 2012, Lilja et al. 2017). These results suggest that Pax7 functions as a pioneer transcription factor. Extensive studies in Pax7 knockout mice indicate that Pax7 regulates multiple aspects of MuSCs. Knocking out Pax7 results in reduced muscle mass, nuclei per myofiber, and diameters of myofibers (Kuang et al. 2006, Oustanina et al. 2004). In MuSCs impairs muscle regeneration and leads to increased fibrosis and accumulation of adipose tissue (von Maltzahn et al. 2013). The defects are more severe after the second round of injury. About 80% reduction of MuSC number was observed in Pax7 knockout mice after continuous injury, suggesting that Pax7 is required for the long-term maintenance of MuSCs (von Maltzahn et al. 2013). Consistently, Pax7 knockout MuSCs display proliferation defect and differentiate prematurely (Gunther et al. 2013, von Maltzahn et al. 2013). Many MuSCs also undergo apoptosis in Pax7 knockout mice, which contributes to the declined MuSC number in Pax7 knockout mice (Relaix et al. 2006). Knocking out Pax7 in Myf5 expressing cells completely blocks muscle regeneration in adult mice, suggesting the crucial role of Pax7 in supporting the expansion of Myf5 + cell (Gunther et al. 2013).

Pax3 is critical for embryonic myogenesis (as reviewed in (Buckingham and Relaix 2015)). Recent studies indicate that it also plays an important role in adult MuSCs. Elevated Pax3 expression promotes MuSC survival and inhibits MuSC differentiation, especially helping MuSC survival while under environmental stress (Crist et al. 2012, Der Vartanian et al. 2019, Hirai et al. 2010). Pax3 and Pax7 share a highly conserved DNA binding domain and recognize the similar DNA sequence. Surprisingly, the DNA binding profiles of Pax3 and Pax7 illustrated by ChIP-seq results show striking differences. Pax3 only binds 6.4% of Pax7 targets (Soleimani et al. 2012). Pax7 tends to recognize and bind homeo box sites, while Pax3 tends to bind paired box elements (Soleimani et al. 2012). These results suggest that Pax3 and Pax7 have distinct functions that are consistent with the observation that Pax3 cannot compensate for the functions of Pax7 in MuSCs (Kuang et al. 2006, Relaix et al. 2006).

A group of transcription factors belonging to the basic Helix-loop-helix protein families critical for myogenesis and named Myogenic Regulatory Factors (MRFs) (Fu et al. 2015a). Myf5 and MyoD are two of them. MyoD (Myogenic determination gene number 1, MyoD1, usually referred to as MyoD) has been regarded as the master transcription factor determining muscle lineage. Ectopic expression of MyoD in many non-muscle cells trans-differentiates them to muscle cells (Davis et al. 1989, Lassar et al. 1989, Weintraub et al. 1989). It forms a heterodimer with E12 or other factors and binds the consensus E box element (As reviewed in (Esteves de Lima and Relaix 2021)). MyoD starts to express after MuSC activation, and its expression persists from proliferating MuSCs to differentiated myotubes (Berkes and Tapscott 2005). The current studies show that binding partners contribute significantly to the selective activation of MyoD target genes. For example, MyoD and FoxO3 mark the super-enhancer in muscle cells, consistent with its role as the master transcription factor of muscle lineage (Peng et al. 2017). At the early stage of MuSC activation and proliferation, MyoD promotes cell proliferation instead of differentiation. How the switch of functions is achieved remains to be answered. Vgll4, an inhibitor of Hippo signaling, participates in the functional transition of MyoD. In proliferating MuSCs, a low dosage of Vgll4 serves as a Hippo signaling inhibitor preventing precocious differentiation. After differentiation induction, the expression level of Vgll4 increases, and Vgll4 can form a complex with TEAD4 and MyoD. The Vgll4-TEAD4-MyoD complex displays higher affinity on Myog promoter binding and activates Myog transcription to promote MuSC differentiation in a Hippo signaling independent manner. Whereas low Vgll4 expression level in proliferating MuSCs is unable to support the formation of Vgll4-TEAD4-MyoD ternary complex (Feng et al. 2019). The selection of MyoD binding partners at various cell status is an interesting question to be answered.

The activity of MyoD is controlled by multiple layers of regulation. Pax3/7 has been shown to activate MyoD transcription in both embryonic and adult myogenesis context (Hu et al. 2008). The location of MyoD gene locus in nuclei also contributes to its transcription regulation mechanism. The MyoD gene locus moves from the nuclear peripheral region to the central nuclear region upon differentiation. The change of nuclear context leads to altered transcription activation of MyoD (Yao and Tjian 2011). The post-translational modification is also important for MyoD activity regulation. MyoD can be phosphorylated by p38-γ on Ser199 and Ser200. The phosphorylated MyoD displays enhanced promoter occupation on the Myog promoter and facilitates Myog transcription to promote MuSC differentiation (Gillespie et al. 2009). R121 of MyoD is subjected to methylation mediated by Protein Arginine Methyltransferase 1 (PRMT1), which also enhances the binding of MyoD on Myog promoter (Liu et al. 2019). Histone deacetylase HDAC1 can demethylate MyoD and inhibit its activity (Mal et al. 2001). CBP/p300 acetylates MyoD to enhance its transcription activator function (Polesskaya and Harel-Bellan 2001). MyoD is also subjected to ubiquitination by HUWE1and MAFbx/AT1 to regulate its half-life in cells (Breitschopf et al. 1998, Noy et al. 2012).

Myf4, Myf5, Myf6, and Myog are other members of MRFs (Fu et al. 2015a). Pax7 activates Myf5 transcription. In Pax7 knockout MuSCs, Carm1 specifically methylates Pax7 at the N-terminus to facilitate the recruitment of H3K4me3 and Mll to the promoter of Myf5 and further activate the transcription of Myf5 (Kawabe et al. 2012). The mRNA of Myf5 exists in quiescent MuSCs, though the protein level of Myf5 is low at this stage. The translation of Myf5 is inhibited by miR31 at the quiescent stage. The protein level increases significantly after MuSC activation due to the reduction of miR31 level (Crist et al. 2012). Myog is activated by MyoD, and it is the key transcription factor to activate the transcription of many genes directly involved in functions of differentiated muscle cells, such as myosin heavy chain (MyHC), myosin light chain (MyLC), and muscle creatine kinase (MCK). Six, myocyte enhancer factor (MEF), and TEAD transcription factor families are also key regulators of MuSC fate change (as reviewed in (Wardle 2019)).

Epigenetic regulation is another key player in myogenesis. As a pioneer transcription factor, Pax7 recruits Trithorax complex to increase the level of H3K4me3 modification on the chromatin and maintain the open chromatin status (Lilja et al. 2017, Soleimani et al. 2012). MyoD can interact with various epigenetic regulatory factors to modulate the transcription of target genes. For example, phosphorylated MyoD recruits Suv39h1/KMT1A to load the repressive H3K9me2 and H3K9me3 marks on chromatin (Robinson and Dilworth 2018).

The dynamic of highly organized chromosome structure is another important regulator of gene expression in MuSCs. Several key transcription factors in myogenesis also play critical roles in modulating the dynamic of 3D chromatin structure. MyoD binding has been shown to be enriched at CCCTC-binding factor (CTCF) binding sites and H3K27Ac regions besides the promoter regions (Dall'Agnese et al. 2019). Hi-C results reveal that MyoD binds topological associated domains (TADs), facilitating promoter-promoter, promoter-enhancer interactions and configuring insulated neighborhoods (Dall'Agnese et al. 2019, Rao et al. 2014). Recently, 3D genome structures in primary muscle cells isolated from mice lacking MyoD versus wild-type mice have been analyzed. MyoD serves as a genome organizer to establish the unique 3D genome architecture in muscle cells. MyoD regulates A/B compartments switch and formation of contact domain boundaries (CDBs) in muscle cells and functions as an anchor protein for myogenic-specific chromatin looping either independent of CTCF or by interacting with CTCF (Wang et al. 2022). MyoD represents one of the best examples of lineage specification transcription factors. The discovery of the “architect role” of MyoD in organizing the cell-type specific structure of the 3D genome implicates that other lineage determination transcription factors may also orchestrate cell-type specific 3D genome organization in diverse organisms (Wang et al. 2022).

During muscle lineage determination, Pax7 enhances the recruitment of active histone marks H3K4me1 and H3K27ac, and increases chromatin accessibility (Lilja et al. 2017). Pax7 binds super-enhancers and works as a key factor for the formation of DNA looping mediating enhancer-promoter interaction. Pax7-dependent DNA looping activates the transcription of MyoD and multiple Myh genes (Zhang et al. 2020). Pax3 recruits LIM domain binding protein 1 (Lbd1) to induce DNA looping and H3K4me1 recruitment in a CTCF-cohesin independent manner. This sub-topologically associated domain interaction is critical for lineage specification (Magli et al. 2019). The role of transcription factors in 3D genome organization in MuSCs and the mechanism of transcription and epigenetic regulation of MuSC activation, proliferation, and differentiation remains to be further investigated.

Non-coding RNAs such as microRNAs (miRs), long non-coding RNAs (LncRNAs), and circular RNAs (circRNAs) have been shown to be part of the epigenetic armamentarium regulating MuSC proliferation and differentiation. There are many elaborated reviews about non-coding RNAs in myogenesis. Here we give a few examples of the studies of non-coding RNAs in epigenetic and transcription regulation.

A subset of miRs enriched in skeletal muscle is named myomiRs, whose expression is controlled by MRFs (Liu et al. 2007). miR-1, miR-133a, miR-133b, miR-206, miR-208b, miR-486, and miR-499 all belong to myomiRs (Horak et al. 2016). Among them, miR-1 and miR-133a are clustered together on the same chromosome. miR206 and miR-133b form another cluster (Nohata et al. 2012). miR-1, miR-133, and miR-206 enhance MuSC differentiation by targeting Pax3, Pax7, HDAC4, Notch3, FGFR1, and PP2AC (Boutet et al. 2012, Chen et al. 2006, Chen et al. 2010, Feng et al. 2013, Gagan et al. 2012, Hindi and Kumar 2016, Liu et al. 2011, Liu et al. 2012). Besides myomiRs, many other miRs such as miR-15b, 22, 24, 27b, 106b, and 431 also regulate MuSC proliferation and differentiation (Mok et al. 2017). miR-195/497, miR-708, miR31, and miR489 participate in the quiescence regulation of MuSCs (Baghdadi et al. 2018b, Cheung et al. 2012, Crist et al. 2012, Sato et al. 2014).

LncRNAs are the other essential important players in MuSC epigenetic regulation. They participate in chromatin structure and status conversion during MuSC fate changes. Lnc-YY1 and maternally expressed 3 (Meg3) can interact with polycomb repressive complex 2 (PRC2) to modify the chromatin status of the target genes and promote MuSC differentiation (Zhou et al. 2015, Zhou et al. 2010). Malat1 inhibits MuSC differentiation by recruiting histone Lysine N-methyl transferase Suv39h1 to load H3K9me3 on MyoD target loci and repress MyoD mediated transcription (Chen et al. 2017). Lnc-MD1 promotes myoblast differentiation by serving as a sponge for miR-133 and miR-135 (Cesana et al. 2011, Gong et al. 2015). Myolinc interacts with TAR DNA binding protein 43 (TDP 43), enhancing TAR 43 recruitment on promoters of MyoD, Myogenin, and other myogenic genes to promote MuSC differentiation (Militello et al. 2018). LncRNA Myoparr is encoded by the promoter of human and mouse Myogenin promoter. Myoparr interacts with transcription co-activator Ddx17 to promote the protein–protein interaction between Ddx17 and histone acetyltransferase p300/CBP associated factor complex (PCAF) and facilitate MuSC differentiation (Hitachi et al. 2019). LncMyo participates in establishing a permissive chromatin environment around E boxes, which enhances MyoD binding and promotes MuSC differentiation (Dong et al. 2020). LncRewind is a recently identified chromatin-associated lncRNA specifically expressed in MuSCs. It interacts with G9a histone lysine methyl transferase to help recruit repressive histone marker H3K9me2 and repress the expression of Wnt7b. LncRewind depletion leads to MuSC differentiation defects (Cipriano et al. 2021).

Transcription factors, co-activators, and co-repressors, together with non-coding RNAs, construct a well-knitted network to regulate the dynamic changes of the epigenetic network in MuSCs. Further analysis of the communications between these components will significantly deepen our understanding on the epigenetic regulation of MuSC fate transition.

## Microenvironment of MuSCs

The microenvironment is an important player in MuSC fate regulation. The quiescent maintenance highly depends on the intact MuSC niche. After muscle injury, the MuSC niche and muscle structure were destroyed. Cells are released from their residency sites and the broken blood vesicles. The components and structure of the extracellular matrix (ECM) are also under significant changes upon injury. All of these make up a transient and complex microenvironment at the lesion. Due to the destruction of the niche, MuSCs are exposed to more cellular signals and have contacts with more cell types. These events are essential cues to regulate MuSC fate.

At the injury site, many damaged myofibers die. It is the early event in muscle regeneration. The damaged myofibers commit a form of programmed cell death named necroptosis. Different from apoptosis, the cell components remain intact and are released after cell membrane eruption. The necroptotic myofibers upregulate the expression of Tenascin C (TNC). The newly made TNC is released to the injury microenvironment. The EGF-like domain at the N-terminus of TNC works as a decoy to activate EGFR signaling by mimicking EGF. The activation of EGFR signaling promotes MuSC proliferation. These “death towards new life” events during muscle regeneration reveal an elaborated strategy evolved in muscle regeneration to make full use of every cell in the injury microenvironment to regulate MuSC proliferation (Zhou et al. 2020).

The cell debris and the cell content leakage from the necroptotic myofibers trigger inflammatory reactions. The complementary system, neutrophils, and mast cells are among the first wave of immune cells being recruited to the lesion. The presence of these immune cells increases the degradation of the injured myofibers, temporarily worsens the muscle injury, and enhances inflammation (Yang and Hu 2018a, Yang et al. 2018b). Next, M1 macrophages infiltrate the lesion. They secrete TNF-α, IL1β, IL6, IL12, inducible nitric oxide synthase (iNOS), and osteopontin promoting the activation and proliferation of MuSCs. The activated T cells are further recruited to the local injury site after the wave of M1 macrophages infiltration (Yang and Hu 2018a, Yang et al. 2018b). The activated T cells secreted TNFα, IFNγ, IL1α, and IL13 to activate the proliferation of MuSCs and promote muscle regeneration. After being treated with the combination of these four factors, MuSCs are capable of long-term expansion in vitro, maintaining their abilities to repair muscle injury, home to the right niche, and support regeneration for multiple rounds of injuries. The activation of T cells is required for muscle regeneration since immune-deficient mice lacking T cells display impaired muscle regeneration. Supplement of the combination of TNFα, IFNγ, IL1α, and IL13 rescues the muscle regeneration defects of immune-deficient mice lacking T cells, suggesting that these four cytokines are the major effective factors secreted by activated T cells to stimulate MuSC proliferation (Fu et al. 2015b).

After the peak of inflammatory reaction, the immune reactions quiet down. Regulatory T cells (Tregs) infiltrate to the injury site and become the primary player in repressing inflammation. Tregs is able to promote MuSC differentiation at the late stage of muscle regeneration (Cho et al. 2019, Wang et al. 2020). Treg cells promote the conversion of pro-inflammatory M1 macrophages to anti-inflammatory M2 macrophages (Schiaffino et al. 2017). M2 macrophages promote MuSC differentiation. Reduction of the number of M2 macrophages leads to impaired muscle regeneration (Wang et al. 2014). In aged mice, decreased IL33 level is correlated with the migration defect of Treg cells into muscle. Supplementation of IL33 restores Treg infiltration and improves muscle injury repair (Yang and Hu 2018a, b).

Microenvironment components also include many metabolic products. The slow twisted myofibers secrete granulocyte colony stimulating factor (G-CSF) enhancing the asymmetric MuSC division (Li et al. 2019). Lactate is a metabolic product of glycolysis. AMPKα1 represses the activity of lactate dehydrogenase (LDH), which catalyzes the pyruvate to lactate conversion. In AMPKα1 knockout mice, the concentration of lactate increases, which promotes MuSC proliferation and inhibits differentiation. While the concentration of lactate is low in MuSCs, it promotes MuSC differentiation (Theret et al. 2017).

Pyruvate Dehydrogenase (PDH) is an enzyme located in mitochondria catalyzing the conversion of pyruvate to acetyl-CoA. It links metabolism to epigenetic regulation in MuSCs And serves as a rheostat for histone acetylation. When PDH level is high, the level of Acetyl-CoA is elevated to generate more histone acetylation on genes related to proliferating to activate their transcription. This feedback supports the continuous proliferation of MuSCs. Consistently, the expression level of PDH is high in proliferating MuSCs, while the abnormally high level of PDH inhibits MuSC differentiation (Yucel et al. 2019).

How the cell fate of MuSCs is regulated by metabolism is now at the early stage of the investigation. Further exploration combining single-cell metabolite analysis, single-cell sequencing, and other techniques will help gain more insight into it.

## Conclusions and perspectives

Skeletal muscle is an organ with critical metabolic functions for the organism and striking regeneration abilities. It represents an excellent model system to study the adult stem cell identity maintenance, cell fate determination, and aging. The works accumulated over the past several decades have revealed many unexpected mechanisms governing the transition between quiescence, proliferation, and differentiation of MuSCs. With the new techniques fitting single cell and single molecule analysis, more insights into the mechanism of cell fate determination will be illustrated. Using MuSCs to treat muscule dystrophy has been proposed and dreamed of for several decades. However, obtaining sufficient amounts of functional MuSCs in vitro has been one of the major obstacles hampering the application of MuSCs in clinic (Montarras et al. 2005). By mimicking the endogenous microenvironment in vitro, functional MuSCs have been expanded in vitro. It paves the road towards the application of MuSCs in regenerative medicines to treat muscular diseases (Fu et al. 2015b). However, we know little about human MuSCs thus far. More investigations about features of human MuSCs are urgently needed. The survival, homing, self-renewal, differentiation, and aging of the transplanted human MuSCs in the recipients are calling for more investigations that will be critical to develop MuSC-based cell therapies for various muscle diseases.

## Data Availability

Not applicable.

## Abbreviations

Ang1: Angiopoietin 1
CalcR: Calcitonin Receptor
CDBs: Contact domain boundaries
circRNAs: Circular RNAs
CTCFCCCTC: Binding factor
CTX: Cardiotoxin
DMD: Duchenne muscular dystrophy
ECM: Extracellular matrix
FoxO: Forkhead box O
G-CSF: Granulocyte colony stimulating factor
HDAC: Histone deacetylase
iNOS: Inducible nitric oxide synthase
ITGB1: Integrinβ1
Jak-STAT: Janus kinase/signal transducer and activator of transcription
Lbd1: LIM domain binding protein 1
LDH: Lactate dehydrogenase
LncRNAs: Long non-coding RNAs
MCK: Muscle creatine kinase
MEF: Myocyte Enhancer Factor
Meg3: Maternally expressed 3
miRs: MicroRNAs
MRFs: Myogenic Regulatory Factors
MuSCs: Muscle stem cells
MyHC: Myosin heavy chain
MyLC: Myosin light chain
OSMβ: Osmotically inducible lipoprotein β
PCAFp300/CBP: Associated factor complex
PDH: Pyruvate Dehydrogenase
PRC2: Polycomb repressive complex 2
PRMT1: Protein Arginine Methyltransferase 1
ROS: Reactive oxygen species
TADs: Topological associated domains
TDP 43: TAR DNA binding protein 43
Tenm4/Odz4: Teneurin transmembrane protein 4
TNC: Tenascin C
Tregs: Regulatory T cells
VEGFA: Vascular Endothelial Growth Factor A
YAP: Yes-associated protein
